# *N*-[(*E*)-4-Bromo­benzyl­idene]-2,3-di­methyl­aniline

**DOI:** 10.1107/S1600536813008088

**Published:** 2013-04-05

**Authors:** Li-Xia Sun, Ling-Zhi Zhu, Jun-Kai Wang

**Affiliations:** a College of Materials Science & Engineering, China Jiliang University, Hangzhou 310018, People’s Republic of China

## Abstract

The title compound, C_15_H_14_BrN, has an *E* conformation about the C=N bond and the dihedral angle between the benzene rings is 50.7 (2)°. In the crystal, mol­ecules are linked *via* C—H⋯π inter­actions, forming columns propagating along [010].

## Related literature

Schiff base derivativies have many pharmaceutical activities. For their anti­fungal effects, see: Aziz *et al.* (2010 ▶), for their radical scavenging activity, see: Lu *et al.* (2012 ▶), for their inhibition of enzyme activity, see: Schmidt *et al.* (2009 ▶) and for their anti­bacterial activity, see: Shi *et al.* (2010 ▶). For related structures, see: Sun *et al.* (2011*a* ▶,*b* ▶); Guo *et al.* (2011 ▶). For standard bond lengths, see: Allen *et al.* (1987 ▶).
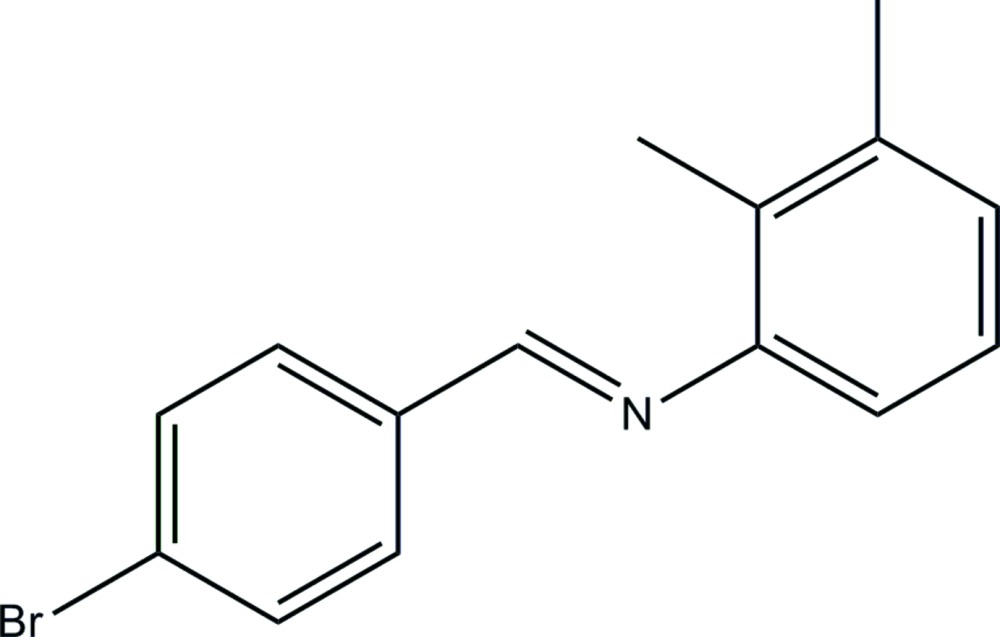


## Experimental

### 

#### Crystal data

C_15_H_14_BrN
*M* *_r_* = 288.18
Monoclinic, 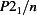

*a* = 12.945 (9) Å
*b* = 7.857 (5) Å
*c* = 14.497 (10) Å
β = 113.384 (8)°
*V* = 1353.3 (16) Å^3^
*Z* = 4
Mo *K*α radiation
μ = 3.02 mm^−1^
*T* = 296 K
0.25 × 0.20 × 0.19 mm

#### Data collection

Bruker APEXII CCD diffractometer
Absorption correction: multi-scan (*SADABS*; Sheldrick, 1996 ▶) *T*_min_ = 0.519, *T*_max_ = 0.598
5865 measured reflections
2525 independent reflections
1154 reflections with *I* > 2σ(*I*)
*R*_int_ = 0.058

#### Refinement

*R*[*F* ^2^ > 2σ(*F* ^2^)] = 0.042
*wR*(*F* ^2^) = 0.112
*S* = 0.94
2525 reflections
157 parameters
H-atom parameters constrained
Δρ_max_ = 0.35 e Å^−3^
Δρ_min_ = −0.25 e Å^−3^

### 

Data collection: *APEX2* (Bruker, 2004 ▶); cell refinement: *SAINT* (Bruker, 2004 ▶); data reduction: *SAINT*; program(s) used to solve structure: *SHELXS97* (Sheldrick, 2008 ▶); program(s) used to refine structure: *SHELXL97* (Sheldrick, 2008 ▶); molecular graphics: *SHELXTL* (Sheldrick, 2008 ▶); software used to prepare material for publication: *SHELXTL*.

## Supplementary Material

Crystal structure: contains datablock(s) global, I. DOI: 10.1107/S1600536813008088/su2578sup1.cif

Structure factors: contains datablock(s) I. DOI: 10.1107/S1600536813008088/su2578Isup2.hkl

Supplementary material file. DOI: 10.1107/S1600536813008088/su2578Isup3.cml

Additional supplementary materials:  crystallographic information; 3D view; checkCIF report

## Figures and Tables

**Table 1:** Hydrogen-bond geometry (Å, °) *Cg*1 and *Cg*2 are the centroids of the C8–C13 and C1–C6 rings, respectively.

*D*—H⋯*A*	*D*—H	H⋯*A*	*D*⋯*A*	*D*—H⋯*A*
C2—H2⋯*Cg*2^i^	0.93	2.99	3.830 (6)	151
C15—H15*B*⋯*Cg*1^ii^	0.96	2.99	3.781 (6)	140

Symmetry codes: (i) 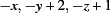; (ii) 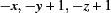.

**Fig. 1.**
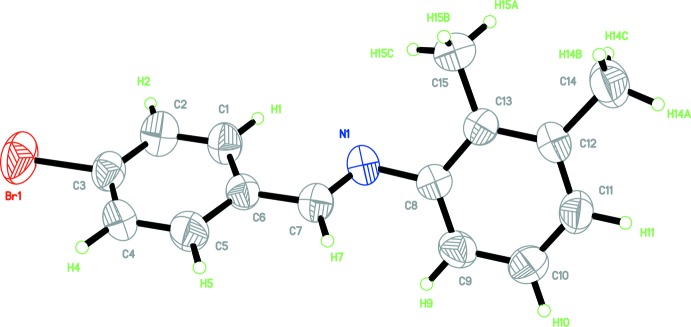
The molecular structure of the title molecule, with atom labelling. The displacement ellipsoids are drawn at the 50% probability level.

## supplementary crystallographic information

### Comment

Schiff base ligands have received much more attention during the past years. They have many pharmaceutical activities, such as antifungal effects (Aziz *et al.*, 2010), radical scavenging activity (Lu *et al.*, 2012), inhibition of enzyme activity (Schmidt *et al.*, 2009), and antibacterial activities (Shi *et al.*, 2010). We report herein on the crystal structure of a new Schiff base compound.

In the title molecule, Fig. 1, the bond lengths (Allen *et al.*, 1987) and angles are normal and comparable to the values observed in similar compounds (Sun *et al.*, 2011*a*,*b*; Guo *et al.*, 2011). The molecule has an E conformation about the C7═N1 bond and is twisted with the dihedral angle between the two aromatic rings being 50.7 (2) °.

In the crystal, molecules are linked via C-H···π interactions (Table 1).

### Experimental

A mixture of 4-bromobenzaldehyde (5 mmol), 2,3-dimethylaniline (5 mmol) and methanol (50 ml) was refluxed for 6 h. The mixture was then allowed to cool and filtered. Recrystallization of the crude product from methanol yielded yellow block-like crystals.

### Refinement

H atoms were positioned geometrically and refined using the riding-model approximation: C—H = 0.93 and 0.96 Å for CH and CH_3_ H atoms, respectively, with *U*_iso_(H) = 1.5*U*_eq_(C-methyl) and = 1.2*U*_eq_(C) for other H atoms.

### Crystal data

**Table:**

C_15_H_14_BrN	*F*(000) = 584
*M**_r_* = 288.18	*D*_x_ = 1.414 Mg m^−^^3^
Monoclinic, *P*2_1_/*n*	Mo *K*α radiation, λ = 0.71073 Å
Hall symbol: -P 2yn	Cell parameters from 2505 reflections
*a* = 12.945 (9) Å	θ = 2.7–25.5°
*b* = 7.857 (5) Å	µ = 3.02 mm^−^^1^
*c* = 14.497 (10) Å	*T* = 296 K
β = 113.384 (8)°	Block, yellow
*V* = 1353.3 (16) Å^3^	0.25 × 0.20 × 0.19 mm
*Z* = 4	

### Data collection

**Table:**

Bruker APEXII CCD diffractometer	2525 independent reflections
Radiation source: fine-focus sealed tube	1154 reflections with *I* > 2σ(*I*)
Graphite monochromator	*R*_int_ = 0.058
φ and ω scans	θ_max_ = 25.5°, θ_min_ = 2.7°
Absorption correction: multi-scan (*SADABS*; Sheldrick, 1996)	*h* = −15→10
*T*_min_ = 0.519, *T*_max_ = 0.598	*k* = −9→7
5865 measured reflections	*l* = −15→17

### Refinement

**Table:**

Refinement on *F*^2^	Secondary atom site location: difference Fourier map
Least-squares matrix: full	Hydrogen site location: inferred from neighbouring sites
*R*[*F*^2^ > 2σ(*F*^2^)] = 0.042	H-atom parameters constrained
*wR*(*F*^2^) = 0.112	*w* = 1/[σ^2^(*F*_o_^2^) + (0.0477*P*)^2^] where *P* = (*F*_o_^2^ + 2*F*_c_^2^)/3
*S* = 0.94	(Δ/σ)_max_ < 0.001
2525 reflections	Δρ_max_ = 0.35 e Å^−^^3^
157 parameters	Δρ_min_ = −0.25 e Å^−^^3^
0 restraints	Extinction correction: *SHELXL97* (Sheldrick, 2008), Fc^*^=kFc[1+0.001xFc^2^λ^3^/sin(2θ)]^-1/4^
Primary atom site location: structure-invariant direct methods	Extinction coefficient: 0.0298 (19)

### Special details

**Table:**

Geometry. All e.s.d.'s (except the e.s.d. in the dihedral angle between two l.s. planes) are estimated using the full covariance matrix. The cell e.s.d.'s are taken into account individually in the estimation of e.s.d.'s in distances, angles and torsion angles; correlations between e.s.d.'s in cell parameters are only used when they are defined by crystal symmetry. An approximate (isotropic) treatment of cell e.s.d.'s is used for estimating e.s.d.'s involving l.s. planes.
Refinement. Refinement of *F*^2^ against ALL reflections. The weighted *R*-factor *wR* and goodness of fit *S* are based on *F*^2^, conventional *R*-factors *R* are based on *F*, with *F* set to zero for negative *F*^2^. The threshold expression of *F*^2^ > σ(*F*^2^) is used only for calculating *R*-factors(gt) *etc*. and is not relevant to the choice of reflections for refinement. *R*-factors based on *F*^2^ are statistically about twice as large as those based on *F*, and *R*- factors based on ALL data will be even larger.

### Fractional atomic coordinates and isotropic or equivalent isotropic displacement parameters (Å^2^)

**Table:**

	*x*	*y*	*z*	*U*_iso_*/*U*_eq_	
Br1	0.07853 (5)	0.88838 (7)	0.11686 (4)	0.0919 (4)	
C1	0.0611 (4)	0.8732 (5)	0.3942 (3)	0.0581 (13)	
H1	0.0091	0.9056	0.4205	0.070*	
C2	0.0362 (4)	0.8999 (5)	0.2939 (3)	0.0643 (13)	
H2	−0.0315	0.9504	0.2529	0.077*	
C3	0.1122 (5)	0.8513 (5)	0.2554 (3)	0.0547 (12)	
C4	0.2111 (4)	0.7767 (7)	0.3137 (4)	0.0736 (15)	
H4	0.2616	0.7431	0.2860	0.088*	
C5	0.2363 (4)	0.7508 (6)	0.4154 (3)	0.0682 (15)	
H5	0.3042	0.7002	0.4557	0.082*	
C6	0.1617 (4)	0.7992 (5)	0.4572 (3)	0.0477 (11)	
C7	0.1882 (4)	0.7675 (5)	0.5637 (3)	0.0513 (12)	
H7	0.2592	0.7258	0.6036	0.062*	
C8	0.1554 (4)	0.7691 (5)	0.7095 (3)	0.0434 (11)	
C9	0.2578 (4)	0.8290 (6)	0.7761 (3)	0.0582 (13)	
H9	0.3060	0.8842	0.7525	0.070*	
C10	0.2887 (4)	0.8071 (7)	0.8781 (3)	0.0713 (14)	
H10	0.3586	0.8452	0.9231	0.086*	
C11	0.2169 (4)	0.7295 (6)	0.9130 (3)	0.0602 (13)	
H11	0.2382	0.7163	0.9818	0.072*	
C12	0.1130 (4)	0.6702 (5)	0.8474 (3)	0.0500 (12)	
C13	0.0802 (3)	0.6918 (5)	0.7437 (3)	0.0449 (11)	
C14	0.0371 (4)	0.5812 (6)	0.8890 (4)	0.0776 (16)	
H14A	0.0750	0.5718	0.9607	0.116*	
H14B	0.0191	0.4695	0.8602	0.116*	
H14C	−0.0309	0.6457	0.8726	0.116*	
C15	−0.0326 (4)	0.6292 (6)	0.6700 (3)	0.0758 (15)	
H15A	−0.0869	0.6384	0.6995	0.114*	
H15B	−0.0262	0.5124	0.6535	0.114*	
H15C	−0.0564	0.6970	0.6100	0.114*	
N1	0.1199 (3)	0.7938 (4)	0.6039 (2)	0.0501 (9)	

### Atomic displacement parameters (Å^2^)

**Table:**

	*U*^11^	*U*^22^	*U*^33^	*U*^12^	*U*^13^	*U*^23^
Br1	0.1350 (6)	0.0914 (5)	0.0529 (3)	−0.0264 (4)	0.0410 (3)	0.0038 (3)
C1	0.064 (3)	0.061 (3)	0.052 (3)	0.015 (3)	0.025 (3)	0.005 (2)
C2	0.075 (4)	0.054 (3)	0.061 (3)	0.021 (3)	0.024 (3)	0.008 (3)
C3	0.069 (3)	0.050 (3)	0.044 (2)	−0.006 (3)	0.023 (3)	0.005 (2)
C4	0.069 (4)	0.104 (4)	0.061 (3)	−0.012 (3)	0.039 (3)	−0.015 (3)
C5	0.051 (3)	0.095 (4)	0.060 (3)	0.013 (3)	0.024 (3)	0.000 (3)
C6	0.051 (3)	0.047 (3)	0.048 (3)	−0.001 (2)	0.023 (2)	−0.001 (2)
C7	0.058 (3)	0.047 (3)	0.050 (3)	0.002 (2)	0.022 (3)	0.000 (2)
C8	0.047 (3)	0.034 (3)	0.049 (3)	0.004 (2)	0.018 (2)	0.001 (2)
C9	0.062 (3)	0.055 (3)	0.063 (3)	−0.015 (3)	0.030 (3)	−0.005 (2)
C10	0.065 (3)	0.089 (4)	0.057 (3)	−0.024 (3)	0.021 (3)	−0.020 (3)
C11	0.072 (4)	0.066 (4)	0.044 (3)	−0.004 (3)	0.024 (3)	−0.002 (2)
C12	0.062 (3)	0.043 (3)	0.053 (3)	0.002 (2)	0.032 (3)	0.003 (2)
C13	0.051 (3)	0.037 (3)	0.046 (2)	0.002 (2)	0.018 (2)	−0.005 (2)
C14	0.093 (4)	0.080 (4)	0.070 (3)	−0.008 (3)	0.042 (3)	0.009 (3)
C15	0.059 (3)	0.100 (4)	0.062 (3)	−0.015 (3)	0.018 (3)	−0.005 (3)
N1	0.061 (2)	0.048 (2)	0.048 (2)	0.004 (2)	0.028 (2)	0.0032 (17)

### Geometric parameters (Å, º)

**Table:**

Br1—C3	1.901 (4)	C8—N1	1.426 (5)
C1—C2	1.375 (6)	C9—C10	1.380 (6)
C1—C6	1.387 (5)	C9—H9	0.9300
C1—H1	0.9300	C10—C11	1.366 (6)
C2—C3	1.365 (6)	C10—H10	0.9300
C2—H2	0.9300	C11—C12	1.383 (6)
C3—C4	1.355 (6)	C11—H11	0.9300
C4—C5	1.393 (6)	C12—C13	1.401 (5)
C4—H4	0.9300	C12—C14	1.513 (6)
C5—C6	1.382 (5)	C13—C15	1.509 (6)
C5—H5	0.9300	C14—H14A	0.9600
C6—C7	1.464 (5)	C14—H14B	0.9600
C7—N1	1.253 (5)	C14—H14C	0.9600
C7—H7	0.9300	C15—H15A	0.9600
C8—C9	1.377 (5)	C15—H15B	0.9600
C8—C13	1.395 (5)	C15—H15C	0.9600
			
C2—C1—C6	121.6 (4)	C10—C9—H9	120.1
C2—C1—H1	119.2	C11—C10—C9	120.1 (4)
C6—C1—H1	119.2	C11—C10—H10	120.0
C3—C2—C1	119.1 (4)	C9—C10—H10	120.0
C3—C2—H2	120.5	C10—C11—C12	121.0 (4)
C1—C2—H2	120.5	C10—C11—H11	119.5
C4—C3—C2	121.5 (4)	C12—C11—H11	119.5
C4—C3—Br1	119.2 (4)	C11—C12—C13	119.6 (4)
C2—C3—Br1	119.3 (4)	C11—C12—C14	119.3 (4)
C3—C4—C5	119.3 (4)	C13—C12—C14	121.0 (4)
C3—C4—H4	120.4	C8—C13—C12	118.5 (4)
C5—C4—H4	120.4	C8—C13—C15	120.3 (4)
C6—C5—C4	120.9 (4)	C12—C13—C15	121.1 (4)
C6—C5—H5	119.6	C12—C14—H14A	109.5
C4—C5—H5	119.6	C12—C14—H14B	109.5
C5—C6—C1	117.7 (4)	H14A—C14—H14B	109.5
C5—C6—C7	120.2 (4)	C12—C14—H14C	109.5
C1—C6—C7	122.1 (4)	H14A—C14—H14C	109.5
N1—C7—C6	123.3 (4)	H14B—C14—H14C	109.5
N1—C7—H7	118.4	C13—C15—H15A	109.5
C6—C7—H7	118.4	C13—C15—H15B	109.5
C9—C8—C13	120.8 (4)	H15A—C15—H15B	109.5
C9—C8—N1	121.1 (4)	C13—C15—H15C	109.5
C13—C8—N1	117.9 (4)	H15A—C15—H15C	109.5
C8—C9—C10	119.9 (4)	H15B—C15—H15C	109.5
C8—C9—H9	120.1	C7—N1—C8	119.4 (4)
			
C6—C1—C2—C3	0.4 (7)	C9—C10—C11—C12	0.6 (8)
C1—C2—C3—C4	0.4 (7)	C10—C11—C12—C13	−0.8 (7)
C1—C2—C3—Br1	−179.8 (3)	C10—C11—C12—C14	178.5 (4)
C2—C3—C4—C5	−0.7 (7)	C9—C8—C13—C12	−2.6 (6)
Br1—C3—C4—C5	179.5 (4)	N1—C8—C13—C12	−178.6 (4)
C3—C4—C5—C6	0.3 (7)	C9—C8—C13—C15	179.0 (4)
C4—C5—C6—C1	0.4 (7)	N1—C8—C13—C15	3.0 (6)
C4—C5—C6—C7	178.6 (4)	C11—C12—C13—C8	1.7 (6)
C2—C1—C6—C5	−0.8 (7)	C14—C12—C13—C8	−177.6 (4)
C2—C1—C6—C7	−178.9 (4)	C11—C12—C13—C15	−179.9 (4)
C5—C6—C7—N1	−173.1 (4)	C14—C12—C13—C15	0.9 (6)
C1—C6—C7—N1	4.9 (7)	C6—C7—N1—C8	−176.2 (4)
C13—C8—C9—C10	2.5 (6)	C9—C8—N1—C7	44.6 (6)
N1—C8—C9—C10	178.4 (4)	C13—C8—N1—C7	−139.3 (4)
C8—C9—C10—C11	−1.5 (7)		

### Hydrogen-bond geometry (Å, º)

Cg1 and Cg2 are the centroids of the C8–C13 and C1–C6 rings, respectively.

**Table:**

*D*—H···*A*	*D*—H	H···*A*	*D*···*A*	*D*—H···*A*
C2—H2···*Cg*2^i^	0.93	2.99	3.830 (6)	151
C15—H15*B*···*Cg*1^ii^	0.96	2.99	3.781 (6)	140

Symmetry codes: (i) −*x*, −*y*+2, −*z*+1; (ii) −*x*, −*y*+1, −*z*+1.
